# The role of the nuclear pore complex in aging of post-mitotic cells

**DOI:** 10.18632/aging.100125

**Published:** 2010-03-02

**Authors:** Martin W. Hetzer

**Affiliations:** Salk Institute for Biological Studies, Molecular and Cell Biology Laboratory, La Jolla, CA 92037, USA

**Keywords:** aging, nuclear pore complex, nucleoporin, intranuclear tubulin, parkinson's disease

The physical separation of the nuclear genome from the cytoplasm by the nuclear envelope (NE) is critical for eukaryotic cell organization. We have discovered that nuclear pore complexes (NPCs), essential multiprotein channels that mediate molecular trafficking across the NE [1], do not turn over and are extremely long-lived in post-mitotic cells [2]. The lack of a replacement mechanism of NPCs leads to a deterioration of NPC function over time, presumably caused by oxidative damage of NPC scaffold components. Age-dependent nuclear pore deterioration is associated with a loss of cell compartmentalization in old cells. This failure of the nuclear permeability barrier is characterized by the leaking of cytoplasmic proteins into the nucleoplasm. We detected large filaments inside the ‘leaky' nuclei of old mouse and rat neurons, which stained with the cytoplasmic protein tubulin [2]. Strikingly, tubulin-positive intranuclear structures have been linked to various neurological disorders including Parkinson's disease [3, 4]. We hypothesize that NPC deterioration might be a general aging mechanism leading to  defects in nuclear function, such as the loss of youthful gene expression programs.

NPCs are multiprotein assemblies that penetrate the nuclear membrane to form aqueous channels across the NE allowing small molecules to freely diffuse between the nucleoplasm and cytoplasm. In contrast, proteins with molecular masses larger than ~60kD are transported through the NPCs by an active, signal-dependent process [5]. NPCs exhibit 8-fold radial symmetry in the plane of the NE and are composed of multiple copies of ~30 different proteins, called nucleoporins (Nups) [6]. Based on their function at the NE, Nups can be classified into (i) scaffold Nups, which mainly consist of the multiprotein Nup107/160 and Nup93/205 complexes [7] and (ii) peripheral Nups. The latter extend from the membrane-embedded scaffold either into the pore channels or as filaments into the cytoplasm or the nucleoplasm [6, 8, 9]. While the scaffold is thought to provide structural integrity to the highly curved pore membrane, the peripheral Nups, many of which contain phenylalanine-glycine (FG)-repeats, are responsible for establishing the permeability barrier [2] and mediating nuclear trafficking [10].

In dividing cells, NPCs disassemble during mitosis and reassemble into the newly forming nuclei. Our recent results suggest that these multi-protein transport channels do not turnover in post-mitotic cells, where the mitotic renewal of NPCs is absent. While peripheral Nups, like Nup153 and Nup50, are continuously exchanged at the NPC, scaffold nucleoporins, like the Nup107/160 complex, are extremely long-lived and remain incorporated in the nuclear membrane during the entire lifespan of a cell. In addition to a lack of nucleoporin expression and NPC turnover, we discovered an age-related deterioration of NPCs leading to a loss of the nuclear permeability barrier and the leaking of cytoplasmic proteins into the nuclear compartment. In the future it will be important to determine the molecular mechanisms that lead to the observed loss of NPC components, determine which cell types are most susceptible for this form of damage and study the physiological consequences of leaky nuclei for cell function such as changes in chromatin organization and gene expression. Our initial studies in the nematode *C. elegans* and rat brain tissue provided evidence that nuclear pore deterioration is linked to oxidative stress [2]. However, it is unclear if NPC components are damaged directly by free radicals or whether loss of NPC components is caused by other mechanisms. For instance, nucleoporins are hyperphosphorylated in mitosis when the entire NPC disassembles [11] and it is possible that aberrant activation of mitotic kinases, which has been observed in adult neurons [11], might result in partial nuclear pore disassembly.

With their highly polarized cell organization, neurons might be particularly sensitive to disruptions in cell compartmentalization. Many signaling proteins and transcription factors shuttle between the nucleus and the cytoplasm and their localization changes in response to different stimuli [12, 13]. In the case of leaky nuclei, these factors might be able to change localization in the absence of a stimulus and thus initiate an aberrant gene expression response. Strikingly, changes in nuclear versus cytoplasmic levels of gene regulatory proteins has been described to occur in old cells [14]. In addition to their role as transport channels, NPCs have been implicated in chromatin organization and gene regulation [4]. For instance, Nup93, a nucleoporin that is damaged and lost during aging, seems to be associated with global histone acetylation [15]. It will also be important to determine if long-lived tissues that experience increased levels of oxidative stress, such as dopaminergic neurons in the substantia nigra, which play a key role in the pathology of Parkinson's disease, are more susceptible to NPC damage.

In summary, the characterization of NPC deterioration at the molecular level might uncover molecular mechanisms that induce or contribute to global changes in genome organization and gene expression in normal and pathological aging.
